# Sensing of C-Reactive Protein Using an Extended-Gate Field-Effect Transistor with a Tungsten Disulfide-Doped Peptide-Imprinted Conductive Polymer Coating

**DOI:** 10.3390/bios12010031

**Published:** 2022-01-07

**Authors:** Kai-Hsi Liu, Hung-Yin Lin, James L. Thomas, Chen-Yuan Chen, Yen-Ting Chen, Chuen-Yau Chen, Chien-Hsin Yang, Mei-Hwa Lee

**Affiliations:** 1 Department of Electrical Engineering, National University of Kaohsiung, Kaohsiung 81148, Taiwan; liukaihsi@gmail.com (K.-H.L.); cychen@nuk.edu.tw (C.-Y.C.); 2 Department of Internal Medicine, Division of Cardiology, Zuoying Branch of Kaohsiung Armed Forces General Hospital, Kaohsiung 81342, Taiwan; 3 Department of Chemical and Materials Engineering, National University of Kaohsiung, Kaohsiung 81148, Taiwan; linhy@ntu.edu.tw (H.-Y.L.); tp65p4m0@gmail.com (C.-Y.C.); 4 Department of Physics and Astronomy, University of New Mexico, Albuquerque, NM 87131, USA; jthomas@unm.edu; 5 Interdisciplinary Program of Electrical Engineering and Computer Science, National Taiwan University of Science and Technology, Taipei 10607, Taiwan; chenyt.daniel@yahoo.com.hk; 6 Department of Materials Science and Engineering, I-Shou University, Kaohsiung 84001, Taiwan

**Keywords:** C-reactive protein, epitope imprinting, tungsten disulfide, electrochemical sensing, human serum

## Abstract

C-reactive protein (CRP) is a non-specific biomarker of inflammation and may be associated with cardiovascular disease. In recent studies, systemic inflammatory responses have also been observed in cases of coronavirus disease 2019 (COVID-19). Molecularly imprinted polymers (MIPs) have been developed to replace natural antibodies with polymeric materials that have low cost and high stability and could thus be suitable for use in a home-care system. In this work, a MIP-based electrochemical sensing system for measuring CRP was developed. Such a system can be integrated with microfluidics and electronics for lab-on-a-chip technology. MIP composition was optimized using various imprinting template (CRP peptide) concentrations. Tungsten disulfide (WS_2_) was doped into the MIPs. Doping not only enhances the electrochemical response accompanying the recognition of the template molecules but also raises the top of the sensing range from 1.0 pg/mL to 1.0 ng/mL of the imprinted peptide. The calibration curve of the WS_2_-doped peptide-imprinted polymer-coated electrodes in the extended-gate field-effect transistor platform was obtained and used for the measurement of CRP concentration in real human serum.

## 1. Introduction 

C-reactive protein (CRP) is a ring-shaped pentameric protein that is found in blood plasma. Elevated serum levels of CRP are associated with inflammation and severe disease in bacterial or viral infections [1]. Low-grade inflammation has recently been discovered to increase the risk of cardiovascular disease [2]. A systemic inflammatory response has been observed in cases of coronavirus disease 2019 (COVID-19), and 97.8% of patients with that disease have CRP concentrations above the normal range [1]. A CRP test may be used to find or monitor conditions that cause inflammation, such as sepsis [3], bowel disease [4], lupus or rheumatoid arthritis [5], and osteomyelitis [6]. CRP has also been implicated in the breakdown of important biological barriers. For example, monomeric CRP can cross the retinal pigment endothelium and may cause macular degeneration through the disruption of the outer blood-retinal barrier [7]. Moreover, C-reactive protein (CRP) has been demonstrated to induce blood brain barrier (BBB) disruption in a process involving NAD(P)H-oxidase dependent oxidative stress [8]. BBB disruption in patients deceased from sepsis was found to correlate with elevated CRP levels [9].

Molecularly imprinted polymers (MIPs) were developed to generate artificial antibodies at low cost and comparatively high stability for the recognition of biomolecules [10]. Two approaches can be used in preparing MIPs that recognize CRP: The whole protein may be used, or specific peptide epitopes derived from the protein sequence. When using the whole protein, it has been important to recognize that the physiological role of CRP is to bind to lysophosphatidylcholine (lysoPC) expressed on the surface of dead or dying cells, and some types of bacteria (by which CRP activates the complement system via C1q [11]). Thus, using functional monomers that mimic lysoPC is important [12,13,14,15]. For example, o-(4-nitrophenylphosphoryl) choline (4NPPC) has been employed as the functional monomer in microcontact imprinting CRP [12], 3(4)-vinylbenzyl 12-phosphorylcholine-dodecanoate was designed and synthesized specifically to form CRP-recognizing MIPs [13]. Notably, the template used in all of the aforementioned approaches was whole CRP, not peptide fragments. When using a peptide epitope, there is no reason to expect that a lysoPC mimic would improve performance (since the peptide alone does not bind lysoPC). In this work, we have taken this approach, using a peptide (KESDTSYVSLKAPL, designated pK) as the imprinted epitope.

**Scheme 1:**
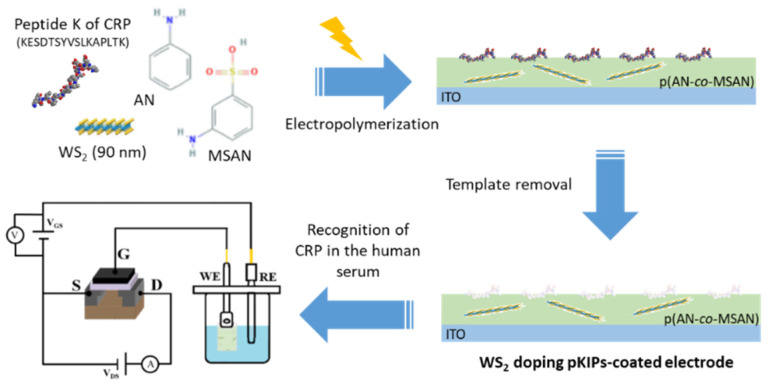
Sensing of C-reactive protein using a tungsten disulfide-doped peptide-imprinted conductive polymer-coated electrode in an extended-gate field-effect transistor.

One decade ago, the chemistry [16], electronic and optoelectronic properties of two-dimensional layered transition metal dichalcogenide (TMDC) nanosheets were reviewed [17]. The structure of the TMDC tungsten disulfide (WS_2_) is based on a hexagonal crystal in which each tungsten (W) atom is six-fold coordinated and hexagonally packed between two trigonally coordinated sulfur (S) atoms [18]. It forms layers that are bound by weak van der Waals’ interactions [16], and these layered structures usually exhibit strongly anisotropic electrical, chemical, mechanical, and thermal properties [17]. Biological sensing [19] and medical diagnosis [20] using TMDCs were both recently reviewed. Tungsten disulfide has also been used in the detection of small molecules (such as H_2_O_2_ and glucose) [20], DNA, and RNA [19]. Our previous studies have demonstrated the utility of doping TMDCs into MIPs for the ultrasensitive determination of 17β-estradiol concentration [21]. TMDCs have also been added to optimize the performance of peptide-imprinted conductive polymers as electrochemical sensors [22]. In the present work, CRP peptide-imprinted polymers were synthesized and optimized using various imprinting concentrations of template peptide molecules and doping concentrations of WS_2_ (Scheme 1). CRP concentrations in real human serum were measured using the extended gate field-effect transistor (EGFET) platform [23,24].

## 2. Results and Discussion 

**Figure 1:**
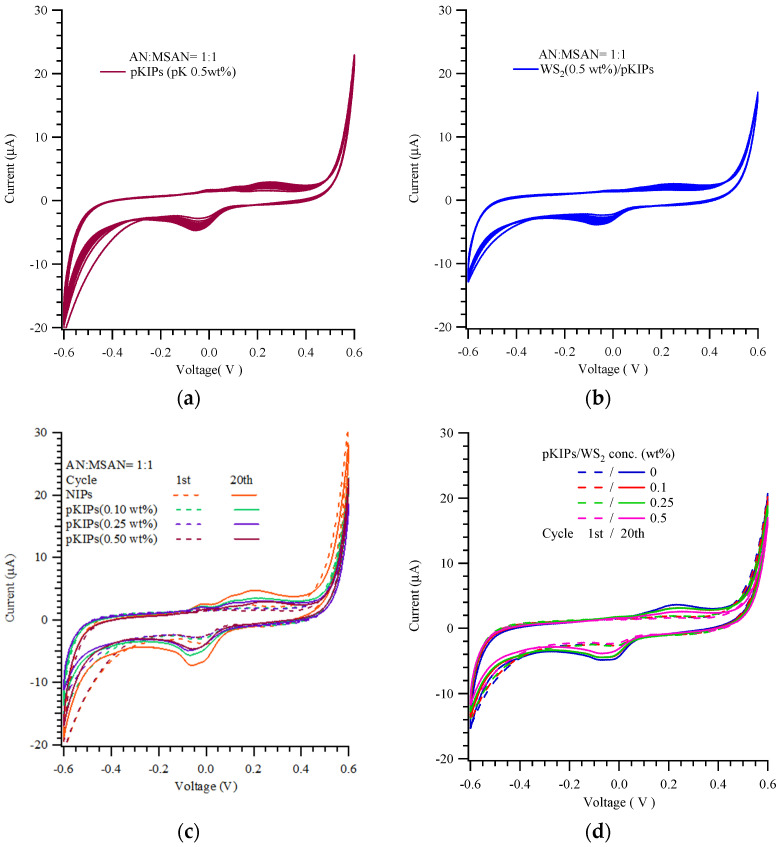
Cyclic voltammetry of peptide K-imprinted polymers (pKIPs), doped with (**a**) 0, (**b**) 0.50 wt% of 90 nm WS_2_ during the electrochemical polymerization. Cyclic voltammetry of the 1st and 20th polymerization cycles during the preparation of peptide-imprinted polymer electrodes with various concentrations of (**c**) peptides K or (**d**) 90 nm WS_2_.

Figure 1a,b presents the electropolymerization curves that show the effects of doping 90 nm WS_2_ flakes into poly(AN-*co*-MSAN), from 0 and 0.5 wt%. There is broad oxidation peak at 0.20–0.25 V and slightly narrower reduction peak at −0.05 V. All curves were with a target pK concentration of 0.5 wt%. The peak currents (both oxidation and reduction) decreased slightly with the increasing concentration of the dopant WS_2_. Figure 1c shows that higher target (pK) concentrations lead to lower polymerization current (at the 20th polymerization cycle). The current intensity of the oxidation peak decreased approximately exponentially as the amount of added peptide increased, as expected when the peptide competes with AN for binding to SO^3−^ on MSAN. Figure 1d shows that higher WS_2_ dopant concentrations have the same effect and also greatly reduce polymerization currents at the 20th cycle. In previous work, we have shown that there is an anticorrelation between polymerization current and sensing current [25]. Therefore, the lower polymerization current should give greater sensing current changes, and thus we selected pK and WS_2_ concentrations of 0.5 wt% for subsequent MIP preparation. Moreover, our previous work also demonstrated the relationship of polymerization duration and the imprinting effectiveness [26] and showed that the thickness of the MIP layer is related to recognition capacity [27].

**Figure 2:**
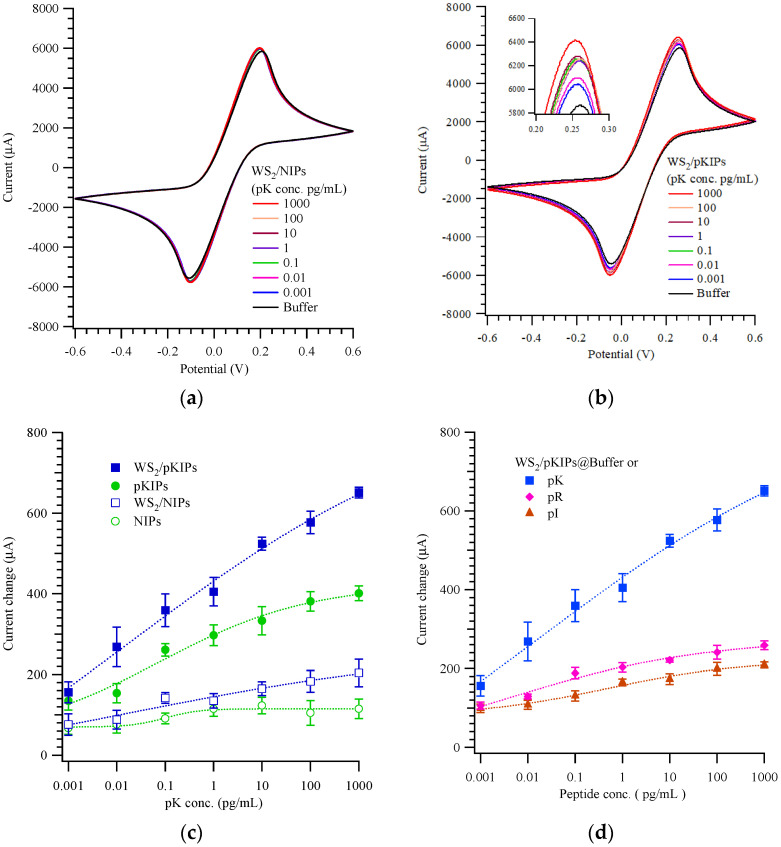
Cyclic voltammograms of various peptide K concentrations using (**a**) WS_2_-doped NIPs- and (**b**) WS_2_-doped pKIPs-coated electrodes. (**c**) Peak oxidation currents (at 0.19~0.21 V), relative to that obtained with buffer, when electrodes were immersed in solutions containing target peptide pK at concentrations varying over seven orders of magnitude, using the electrodes shown in (**a**,**b**). (**d**) Interference of CRP pR and pI peptides on the WS_2_-doped pKIPs-coated electrode.

Figure 2a,b display the cyclic voltammograms of WS_2_/NIPs- and WS_2_/pKIPs-coated electrodes used to sense various peptide K concentrations in an aqueous solution. The oxidation and reduction current peaks were at 0.2 and −0.1 V, respectively, possibly corresponding to the oxidation and reduction of coupled Fe(CN)6^3+^ and Fe(CN)_6_^4+^. Figure 2c shows the calibration curves obtained from the voltammograms in Figure 2a,b. The calibration curves plot the difference between the peak current obtained in a pK (target) solution and that obtained in buffer alone for pK concentrations varying over seven orders of magnitude. The pKIP- and NIP-coated electrodes began to show an onset of saturation (i.e., a leveling-off of the response) beginning at about 1.0 pg/mL, Figure 2c. Doping with WS_2_ increases the amplitude of the response but also extends the (log)-linearity and useful sensing range up to at least 1.0 ng/mL. Figure 2d shows the responses of WS_2_-doped pK-imprinted electrodes to other peptides taken from CRP sequences. The response of the electrode to these irrelevant peptides is very similar to the responses obtained with non-imprinted electrodes to pK. This is an important result that we will discuss further, vide infra.

Figure S1, from top to bottom, displays the surface morphologies of WS_2_/NIPs- (left column) and WS_2_/pKIPs- (right column) coated electrodes before and after template removal and upon rebinding with 1.0 pg/mL of pK. The sizes of aggregates of poly(AN-*co*-MSAN) is approximately 50 to 100 nm. Figure S2a,b plot the electrochemical responses of WS_2_/NIPs- and WS_2_/pKIPs-coated electrodes in buffer, 1.0 pg/mL of peptide K and 1.0 pg/mL of CRP at various scan rates. These were fit using the Randles–Sevcik equation. Interestingly, the gradients of the fitted curves for WS_2_/pKIPs exceed those for WS_2_/NIPs, possibly indicating that the binding of pK or CRP increased the surface area of the electrodes. The larger surface area raises the concern that the higher responses in Figure 2c arise from non-specific binding to a larger surface area. If this were so, irrelevant peptides would also give strong electrochemical signals. However, as Figure 2d shows, irrelevant peptides do not give strong signals with the imprinted electrode, showing that increased surface does not give a larger signal from non-specific binding and that the WS_2_/pKIPs-coated electrodes are selective for the imprinted target. Figure S2c plots the AC impedance measurements of WS_2_-doped pKIPs and NIPs in buffer, peptide K and CRP 1.0 pg/mL solutions. The resistance of pKIPs was almost the same as that of NIPs, but decreased upon the rebinding of pK and CRP. Finally, Figure S2d presents the reusability of the WS_2_/pKIPs-coated electrodes. One of those electrodes was used to measure the relative peak current changes of a 1.0 pg/mL solution of CRP, rinsed, and then reused five times. There was only a small deterioration in the response on the fifth and sixth usage.

**Figure 3:**
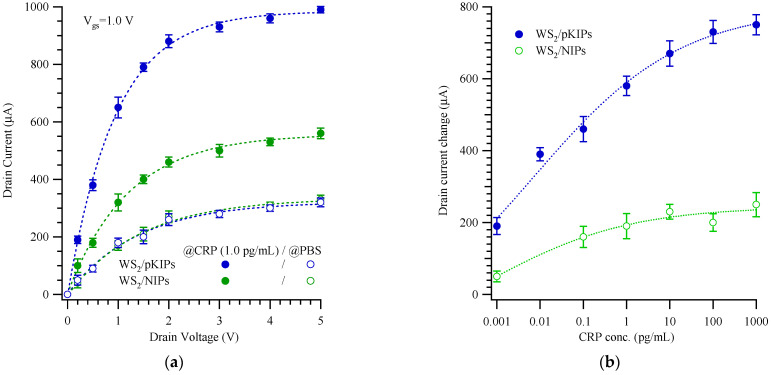
(**a**) Electrochemical response of WS_2_-doped peptide K-imprinted p(AN-*co*-MSAN)-coated electrodes in CRP and PBS with various drain voltages. (**b**) Calibration curves measured with tungsten disulfide doping peptide K-imprinted p(AN-*co*-MSAN)-coated extended-gate field-effect transistor. The voltage between drain or gate and source electrodes are 1.5 and 1.0 V, respectively.

Finally, the pKIP-coated electrode was integrated into an extended-gate FET as shown in Scheme 1. The drain current was measured as a function of drain voltage (Figure 3a) for both WS_2_-doped pKIPs and WS_2_-doped NIPs at 1.0 pg/mL CRP concentration and in buffer alone. By repeating the measurement with several different CRP concentrations, a calibration curve of drain current (above buffer only) vs. CRP concentration was constructed, Figure 3b. Interestingly, the electrochemical response (sensitivity) of pK on WS_2_/pKIP-coated electrodes is higher on the FET platform than in the cyclic voltammogram in Figure 3a. The calibration curve (Figure 3b) on the FET platform was then used to make measurements of the real serum sample. The sensing range is from 1.0 fg/mL to 1.0 ng/mL, which is comparable with the MIPs using PC analogs as functional monomers (e.g., 2-acryl amidoethyldihydrogen phosphate [14] or 4NPPC/polyethylene glycol 400 dimethacrylate [15], which gave sensing ranges of 0.07–8.50 and 5–120 μg/mL, respectively, using differential pulse voltammetry or circular dichroism). Other imprinted CRP sensor approaches include whole CRP imprinted coupled with gold-platinum bimetallic nanomaterials to enhance the sensor’s surface area and catalytic activity. This sensor had a large range (0.1–500 nM or 12 ng/mL–60 μg/mL) but a much higher limit of detection than the sensor in this work [28]. A graphdiyne-based CRP MIPs biosensor performed with a very broad detection range from 10^−5^ to 10^3^ ng/mL [29]. The WS_2_/pKIP-coated electrode in this work outperforms (in sensitivity) a CRP immunosensor (with sensing range 0.01–1000 ng/mL), which uses antibodies to recognize target molecules [30]. Moreover, our recent study shows that the effect of doping of MIPs with a very small percentage of an MXene (e.g., Ti_2_C at 0.1 wt% in the preparation solution) can give an electrochemical sensing range from 0.1 to 10,000 fg/mL [31]. The doping of WS_2_ nanoflakes in this work may further extend the sensing range. Table 1 shows measurements on a real serum sample. A peak current change of 694.2 ± 1.2 μA was found, which converts to a CRP concentration of around 2.28 ± 0.12 μg/mL. The CRP concentration in the serum sample was checked using ELISA, indicating that the pKIP FET measurement had an accuracy of approximately 96%.

## 3. Conclusions

We constructed a sensitive FET-based sensor for CRP, using peptide epitope imprinting (imprinting a peptide rather than a whole protein). This approach obviates the need to use ligand-mimicking functional monomers in the MIP composition. Flaked tungsten disulfide was used as a dopant in the recognition polymer, which was seen to increase both the response and the useful sensing range. The sensor proved to be sensitive and accurate on real serum when compared to ELISA results. Such economic approaches to biomarker sensing may be cost-effective for future home-care diagnostic systems, which can be integrated with a potentiostat [32] and a microfluidic device [33] or for wearable biosensor devices.

## Figures and Tables

**Table 1:** Measurements of CRP in human serum by the WS_2_/pKIPs-coated sensors and ELISA kit. The standard deviations are based on at least three individual measurements.

No.	WS_2_/pKIPs (μA)	Converted conc. (μg/mL)	Average CRP conc. (μg/mL)		Accuracy (%)
			WS_2_/pKIPs	ELISA	
1	694	2.26	2.28 ± 0.12	2.37 ± 0.20	96
2	695	2.36			
3	693	2.16			
4	696	2.46			
5	693	2.16			

## Data Availability

The authors confirm that the data supporting the findings of this study are available within the article and its Supplementary Materials.

## Supplementary Materials

The following are available online at https://www.mdpi.com/article/10.3390/bios12010031/s1, Figure S1: SEM images of 90 nm WS_2_ (0.5 wt%) doped (a) NIPs, and (b) peptide K-imprinted poly(AN-*co*-MSAN)-coated electrodes before template removal (a,b), after template removal (c,d), and after rebinding peptide K (e,f); Figure S2: The electrochemical response of (a) WS_2_/NIPs and (b) WS_2_/pKIPs-coated electrodes in buffer, 1.0 pg/mL of pK or CRP. The responses were then fit with Randles–Sevcik equation for the solution at 25 °C, ip = 268,600 n^3/2^ AD^1/2^Cv^1/2^. (c) AC impedance measurements for WS_2_/NIPs- and WS_2_/pKIPs-coated electrodes in buffer, 1.0 pg/mL of pK or CRP solutions. (d) The reusability of the WS_2_/NIPs and WS_2_/pKIPs-coated electrodes. The electrode was used to measure a 1.0 ng/mL solution of CRP, rinsed and reused for five cycles.
