# Supplementary material for: Sensing of C-Reactive Protein Using an Extended-Gate Field-Effect Transistor with a Tungsten Disulfide-Doped Peptide-Imprinted Conductive Polymer Coating
Source: Biosensors (Basel). 2022 Jan 7;12(1):31. doi: 10.3390/bios12010031 (PMC8774123; doi:10.3390/bios12010031)
Supplement: Supplementary file 1 [file biosensors-12-00031-s001.zip › biosensors-1502302-supplementary.pdf]

biosensors

MDPI

Supporting Information:

Sensing of C-Reactive Protein Using an Extended-Gate Field-Effect Transistor with a Tungsten Disulfide-Doped Peptide-Imprinted Conductive Polymer Coating

Kai-Hsi Liu [1,2,†], Hung-Yin Lin [3,†], James L. Thomas [4], Chen-Yuan Chen [3], Yen-Ting Chen [5], Chuen-Yau Chen [1], Chien-Hsin Yang [3,*] and Mei-Hwa Lee [6,*]

[1] Department of Electrical Engineering, National University of Kaohsiung, Kaohsiung 81148, Taiwan; liukaihsi@gmail.com (K.-H.L.); cychen@nuk.edu.tw (C.-Y.C.)
[2] Department of Internal Medicine, Division of Cardiology, Zuoying Branch of Kaohsiung Armed Forces General Hospital, Kaohsiung 81342, Taiwan
[3] Department of Chemical and Materials Engineering, National University of Kaohsiung, Kaohsiung 81148, Taiwan; linhy@ntu.edu.tw (H.-Y.L.); tp65p4m0@gmail.com (C.-Y.C.)
[4] Department of Physics and Astronomy, University of New Mexico, Albuquerque, NM 87131, USA; jthomas@unm.edu
[5] Interdisciplinary Program of Electrical Engineering and Computer Science, National Taiwan University of Science and Technology, Taipei 106, Taiwan; chenyt.daniel@yahoo.com.hk
[6] Department of Materials Science and Engineering, I-Shou University, Kaohsiung 84001, Taiwan
* Correspondence: yangch@nuk.edu.tw (C.-H.Y.); meihwalee@ntu.edu.tw (M.-H.L.)
† These authors contributed equally to this work.

Citation: Liu, K.-H.; Lin, H.-Y.; Thomas, J.L.; Chen, C.-Y.; Chen, Y.-T.; Chen, C.-Y.; Yang, C.-H.; Lee, M.-H. Sensing of C-Reactive Protein Using an Extended-Gate Field-Effect Transistor with a Tungsten Disulfide-Doped Peptide-Imprinted Conductive Polymer Coating. *Biosensors* **2022**, *12*, 31. https://doi.org/10.3390/bios12010031

Received: 25 November 2021
Accepted: 3 January 2022
Published: 7 January 2022

Publisher's Note: MDPI stays neutral with regard to jurisdictional claims in published maps and institutional affiliations.

Copyright: © 2022 by the authors. Licensee MDPI, Basel, Switzerland. This article is an open access article distributed under the terms and conditions of the Creative Commons Attribution (CC BY) license (http://creativecommons.org/licenses/by/4.0/).

S1. Experimental

S1.1 Reagents.

Three C-reactive protein peptides, KESDTSYVSLKAPL (pK), ICTSWESASGIVEF (pI), RALKYEVQGEVFTK (pR), were ordered from Yao-Hong Biotechnology Inc. (HPLC grade, New Taipei City, Taiwan). Aniline (AN), tungsten disulfide (WS_2 90 nm avg. particle size (SEM)), recombinant C-reactive protein (CRP, #C1617) expressed in *E.* coli, and human serum (#H4522-20ML) from human male AB plasma that was of USA origin and sterile-filtered were acquired from Sigma-Aldrich Co. (St. Louis, MO). *M*-Aminobenzenesulfonic acid (MSAN) was acquired from Acros Organics, New Jersey). All chemicals were used as received unless otherwise mentioned.

S1.2 Electropolymerization of WS_2-doped peptide K-imprinted poly(AN-co-MSAN)s on electrodes.

The peptide KESDTSYVSLKAPL (pK) was added to an equimolar solution of AN and MSAN at various concentrations (0-0.5 wt%) in 57 mM of DI water, as shown in Scheme 1 [1]. Indium tin oxide (ITO) glasses (from RuiLong glass in 200x 370x 0.7 mm^3, with surface resistivity 7 Ω/sq) were cut to 1.0 × 2.0 cm^2 in size and were immersed in the monomer/WS_2/template mixture for electropolymerization (1.0x 1.0 cm^2). Tungsten disulfide nanoflakes were then added to the monomer mixtures at concentrations of 0-0.5 wt% to prepare the WS_2-doped peptide-imprinted or non-imprinted poly(AN-*co*-MSAN)-coated electrodes. These electrodes were then washed in 10 mL of 5 vol% ethanol at 130 rpm for 10 min on an orbital shaker (OSR201-1, GenePure technology, Taichung, Taiwan), and this procedure was repeated using a pure DI water wash. The same washing procedure was used to remove any bound CRP during the reusability studies. The electrodes were then connected to a potentiostat (CHI 400C, CH Instruments, Inc., Austin, TX), and cyclic potential (−0.6 to 0.6 V vs. Ag/AgCl at 0.1 V/s scan rate) was applied [1]. The counter and reference electrodes were platinum wire and Ag/AgCl (RE-1B, ALS Co. Ltd., Tokyo Japan).

S1.3 Characterization of peptide K-imprinted poly(AN-co-MSAN) conductive films with and without doping with WS_2 nanoflakes.

The electrochemical reactions between the target molecules and electrodes were controlled and monitored with a potentiostat (608-1A, CH Instruments, Inc., Austin, TX). The working, counter, and Ag/AgCl reference electrodes were immersed in a 20 mL solution containing 125 mM KCl, 5 mM $K_4Fe(CN)_6$, and 5 mM $K_3Fe(CN)_6$. The potential was scanned from -0.6 V to 0.6 V at 100 mV/s unless otherwise mentioned, and the effects of the peptides or proteins on the peak currents for the ferri-/ferrocyanide system were recorded [2,3]. AC impedance was characterized (ZENNIUM/IM6, Zahner-electrik GmbH& Co KG) for electrodes coated with WS_2/pKIP or NIPs/WS_2 [4]. Measurements were made with buffer, 1.0 pg/mL of pK, or CRP.

S1.4 CRP measurements in diluted human serum samples on the FET platform

Scheme 1 shows the setup of the FET platform. For the MOS transistor (CD4007UBE, Texas Instruments Incorporated, Dallas, Texas, USA), the gate-to-source voltage V_{GS} was applied at a constant voltage source of 1.0 V generated by the dc power supply (GPC-30300, GW Instek, Taipei, Taiwan) in series with the potential difference between the WE and RE electrodes; the drain-to-source voltage V_{DS} was applied by a voltage source V_D generated by the dc power supply (GPC-30300, GW Instek, Taipei, Taiwan), with voltages ranging from 0.5 V to 5 V and increasing at0.5 V increments every 10 minutes. The constant voltage source $V_G = 1.0\,\mathrm{V}$ prevented the MOS transistor from being biased at the cut-off region. The potential difference between WE and RE heavily depends on two factors: the doping conditions of the electrodes and the concentration of the solution. The gate-to-source voltage V_{GS} of the MOS transistor was adjusted accordingly based on the specific doping level of the electrodes and on the concentration of the solution. Therefore, we were able to measure the drain-to-source current I_{DS} of the MOS transistor with a digital multimeter (GDM-7145, GW Instek, Taipei, Taiwan) and complete the transfer characteristics resulting from the triode region to the saturation region of the MOS transistor for specific electrode-doping conditions or solution concentrations.

The peptide K-imprinted poly(AN-*co*-MSAN)-coated electrode was used as WE on the FET platform to measure different concentrations of CRP in 125 mM KCl, 5mM $K_4Fe(CN)_6$, and 5 mM $K_3Fe(CN)_6$ buffer solution to construct a calibration curve based on the Hill equation (Fig. 3). Samples were incubated with the electrode for 10 minutes to reach equilibrium. Human serum from human male AB plasma was diluted into the same buffer for real samples. Human C-reactive protein enzyme-linked immunosorbent assay (ELISA) kit (#KHA0031, Invitrogen - Thermo Fisher Scientific) was purchased for the human male AB plasma measurements, and the protocol from the product sheet was followed (https://www.thermofisher.com/elisa/product/CRP-Human-ELISA-Kit/KHA0031).

S1.5 Data Analysis

All experiments were carried out in triplicate, and data are expressed as means ± standard deviation.

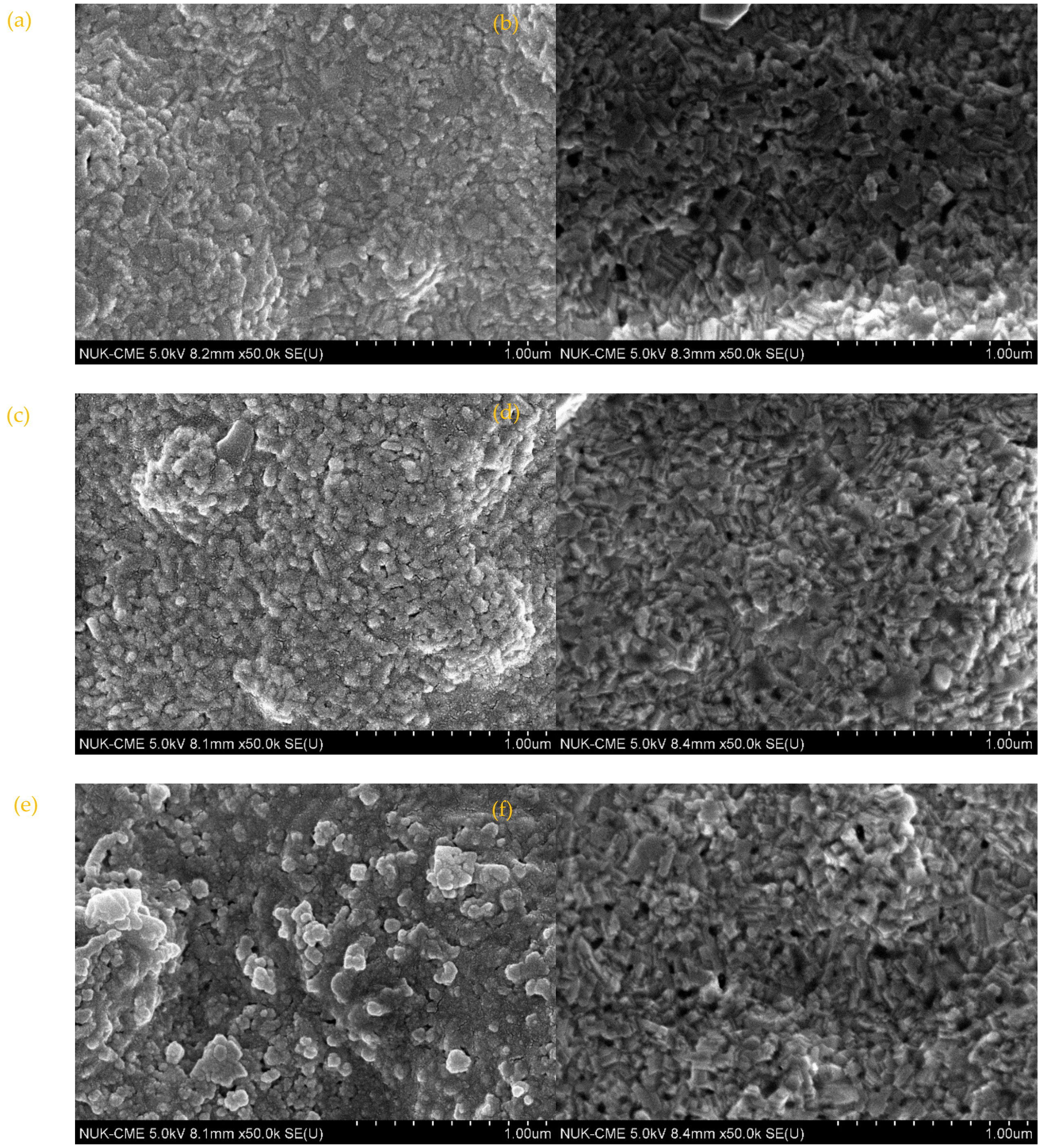

Figure S1. SEM images of 90 nm WS_2-(0.5 wt%) doped (a) NIPs and (b) peptide K-imprinted poly(AN-*co*-MSAN)-coated electrodes before template removal ((a) and(b)), after template removal ((c) and(d)), and after rebinding peptide K ((e) and (f)).

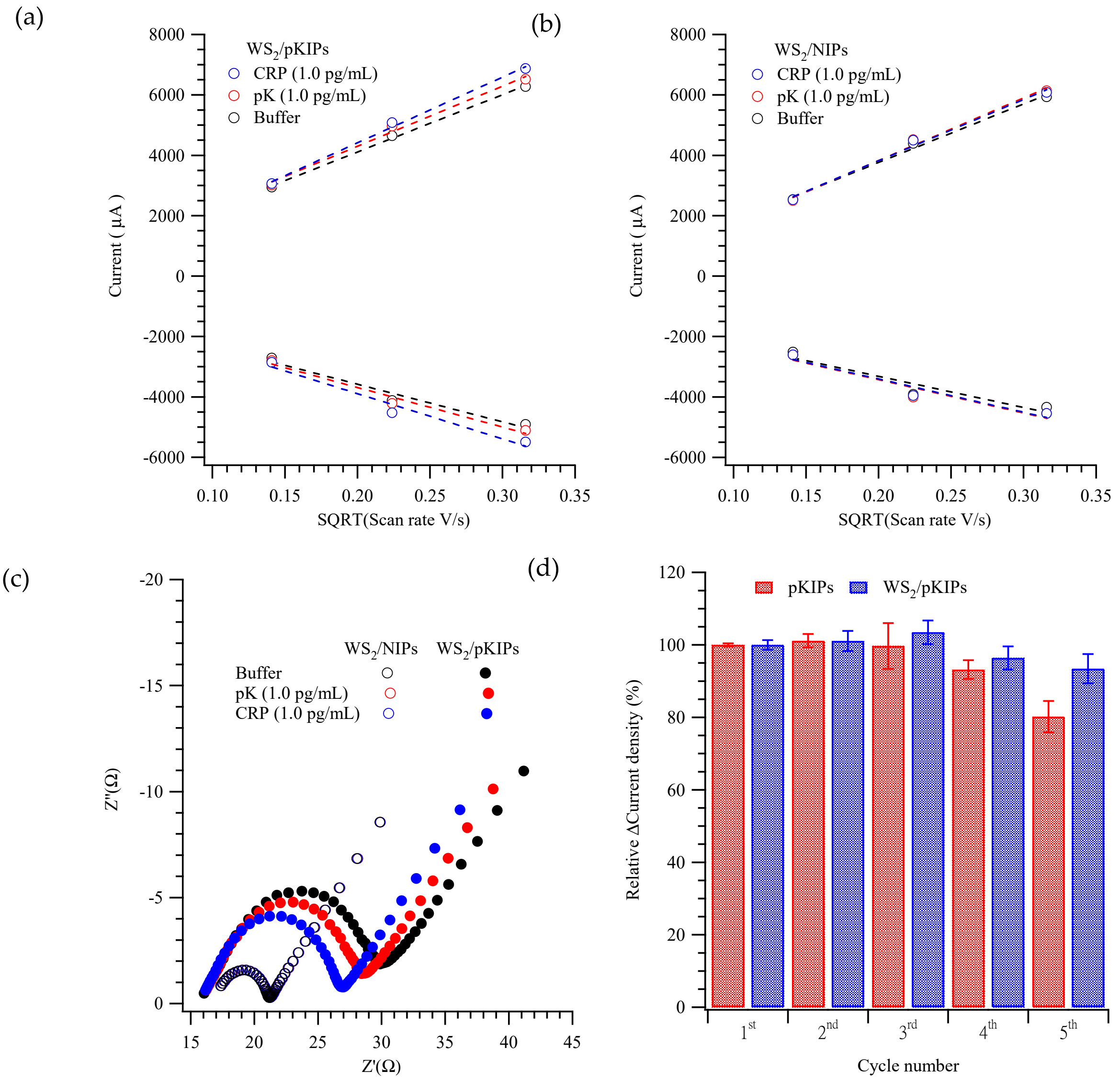

Figure S2. The electrochemical response of (a) WS_2/NIPs and (b) WS_2/pKIPs-coated electrodes in buffer, 1.0 pg/mL of pK, or CRP. The responses were then fit using the Randles–Sevcik equation for the solution at 25°C, i_p=268600$n^{3/2}$ $AD^{1/2}Cv^{1/2}$. (c) AC impedance measurements for WS_2/NIPs- and WS_2/pKIPs-coated electrodes in buffer, 1.0 pg/mL of pK, or CRP solutions. (d) The reusability of the WS_2/NIPs and WS_2/pKIPs-coated electrodes. The electrode was used to measure a 1.0 ng/mL solution of CRP and was then rinsed and reused for five cycles.

References

1. Lee, M.-H.; O'Hare, D.; Guo, H.-Z.; Yang, C.-H.; Lin, H.-Y. Electrochemical sensing of urinary progesterone with molecularly imprinted poly(aniline-co-metanilic acid)s. *Journal of Materials Chemistry B* **2016**, *4*, 3782-3787, doi:10.1039/C6TB00760K.
2. Huang, C.-Y.; Tsai, T.-C.; Thomas, J.L.; Lee, M.-H.; Liu, B.-D.; Lin, H.-Y. Urinalysis with molecularly imprinted poly(ethylene-co-vinyl alcohol) potentiostat sensors. *Biosensors and Bioelectronics* **2009**, *24*, 2611-2617.

3. Huang, C.-Y.; O'Hare, D.; Chao, I.J.; Wei, H.-W.; Liang, Y.-F.; Liu, B.-D.; Lee, M.-H.; Lin, H.-Y. Integrated potentiostat for electrochemical sensing of urinary 3-hydroxyanthranilic acid with molecularly imprinted poly(ethylene-co-vinyl alcohol). *Biosensors and Bioelectronics* **2015**, *67*, 208-213, doi:http://dx.doi.org/10.1016/j.bios.2014.08.018.
4. Lee, M.-H.; Thomas, J.L.; Su, Z.-L.; Zhang, Z.-X.; Lin, C.-Y.; Huang, Y.-S.; Yang, C.-H.; Lin, H.-Y. Doping of transition metal dichalcogenides in molecularly imprinted conductive polymers for the ultrasensitive determination of 17β-estradiol in eel serum. *Biosensors and Bioelectronics* **2020**, *150*, 111901, doi:https://doi.org/10.1016/j.bios.2019.111901.
